# Barriers and facilitators to bidirectional screening of TB-DM in Ghana: Healthcare workers’ perspectives

**DOI:** 10.1371/journal.pone.0235914

**Published:** 2020-07-14

**Authors:** Rita Suhuyini Salifu, Khumbulani Welcome Hlongwana

**Affiliations:** 1 Discipline of Public Health Medicine, School of Nursing and Public Health, University of KwaZulu-Natal, Durban, South Africa; 2 Health and Development Solutions Network, Tamale, Ghana; York University, CANADA

## Abstract

**Background:**

The tuberculosis (TB) and diabetes mellitus (DM) co-epidemic continues to increase globally. Low-and middle-income countries bear the highest burden of co-epidemic, and Ghana is no exception. In 2011, the World Health Organisation (WHO) responded to this global challenge by launching a collaborative framework with a view to guide countries in implementing their DM and TB care, prevention and control plans. Subsequently, several countries, including Ghana, adopted this framework and began implementing bidirectional screening of TB and DM patients. Almost a decade later since the launch of the framework, the implementation of bidirectional screening in Ghana has not been subjected to empirical research. This study explored the barriers and facilitators to bidirectional screening through the lenses of the implementing healthcare workers.

**Methods:**

This was an exploratory qualitative study conducted in three public health facilities offering both TB and DM services in Northern Ghana. In-depth interviews, document review and observations, were used to generate data. In total twenty-three healthcare workers (doctors, nurses, prescriber, health managers and TB task- shifting officers delivering care in TB and DM clinics) were interviewed, using semi-structured interview guides. The interview questions solicited information on the screening process, including knowledge of the collaborative framework, comorbidity, collaboration and workload.

**Results:**

Six themes emerged from the analysis, of which two (Increase in staff capacity, and Institutionalisation of bidirectional screening) were facilitators, and four (Delays in screening, Fear and stigmatization of TB, Poor collaboration between TB and DM units, and Skewed funding for screening) were barriers.

**Conclusions:**

The implementation of bidirectional screening at public health facilities in Ghana was evident in this study and increased staff capacity, funding and institutionalisation enhanced the policy implementation process. However, the screening of TB patients for DM is yet to be prioritised, and emphasis should be put on the design for cost-effective screening approaches for low- and middle-income countries.

## Introduction

Globally, the linkage between diabetes mellitus (DM) and tuberculosis (TB) has been well established [1]. People living with DM are three times more likely to develop TB, and an increase in DM predisposes people to develop TB [2, 3]. In 2013, the worldwide TB-DM comorbidity was over 1 million cases, reflecting 15% of the global tuberculosis burden [4, 5]. Studies conducted in various countries from 2011–2013 found DM prevalence in TB patients to be from 25%-44% between 2011–2012 in three regions of India, 36% in Mexico, 17% in Tanzania and 40–45% in the South Pacific [5, 6]. Projections indicate that by 2045, 629 million people will be living with DM, and the greatest share will be borne by LMICs [2]. If the situation is not averted, the double burden of DM and TB is expected to adversely affect the numerous Stop TB global initiatives, which were first initiated through the Millennium Development Goals (MDGs) and rolled over to the Sustainable Development Goals (SDGs) with an ultimate goal of creating “A world free of tuberculosis” [7]. The urgency to address this co-epidemic is fuelled by the bleak picture painted in current research estimates and future projections showing that there are more people living with TB-DM comorbidity than TB-HIV comorbidity, worldwide, a trend that is likely to grow [5, 8].

The extent of this double burden of TB-DM disease on LMIC populations and how health systems have adapted and cope with this burden is yet to be fully understood [9]. The 2019 World Health Organisation (WHO) Global TB Report maintains that TB is still among the top ten causes of mortality worldwide and a single infectious agent causing more mortality even above HIV/AIDS [10]. Ghana is an LMIC where TB is still a major contributor to morbidity and mortality. The national TB incidence is 148/100,000 population; though as many as 2/3 of the country’s cases are estimated to be missed due to inadequate or misdiagnoses [10, 11].TB prevalence is found to increase with age among the adult population [10]. The national prevalence of DM is also high—3.6% in adult population, and Ghana continues to face challenges relating to undiagnosed DM cases, which in turn leads to increased complications with assessing outcomes for DM-TB [12].

To mitigate the co-epidemic of TB and DM, the WHO and International Union Against Tuberculosis and Lung Disease (The Union) have developed a collaborative framework for the care and control of DM and TB [3]. A key component of this collaborative framework is screening TB patients for DM and DM patients for TB, or bidirectional screening [3]. Since its launch in 2011, health systems of various countries, including Ghana, have adopted the collaborative framework and are at various stages of implementation[5]. Ghana’s NTP begun the pilot phase of the Intensive Case Finding Initiative (ICF) between 2009–2013 and has since expanded it to all facilities offering TB care [11]. The objective of the pilot phase was to assess the feasibility of screening all patients who visit public health facilities for TB [11]. However, beyond the pilot phase, there is a paucity of empirical evidence on the implementation of bidirectional screening in Ghana [11]. This research was aimed at exploring the barriers and facilitators to bidirectional TB and DM screening at three healthcare facilities in Northern Ghana, from the perspectives of the implementing healthcare workers, using qualitative inquiry.

## Materials and methods

### Setting of the study

This study was conducted in the Northern Region (NR) of Ghana, a country located in West Africa [13]. The NR is one of 16 administrative regions with a population of 1,905,628 and largely rural dispersed settlements [14, 15]. According to estimates from 2016, Ghana poverty and inequality report, 50.4% of the NR population live in poverty, compared to the 5.6%-14.8% for the rest of Ghana, thus making the NR the highest poverty-trapped region in the country [16]. The poverty profile of the region and long distances between settlements poses a challenge in healthcare delivery and health-seeking behaviour of patients[17]. This study took place in three public health facilities that offer both TB and DM care [15]. The names of these three facilities are withheld for privacy and confidentiality purposes.

### Study design

This research used an exploratory qualitative design to explore barriers and facilitators to the bidirectional TB and DM screening process in Ghana [18]. Twenty-three (23) healthcare workers involved in TB and DM care were recruited using a heterogenous purposive sampling technique to ensure that diverse perspectives were captured. Sampled healthcare workers were heterogeneous in terms of professions, age, gender and roles. To ensure rigor, data generated were further triangulated through comparing and contrasting information generated from the in-depth interviews with observational data and document reviews [19].

### Data generation

Data for this study were generated over three (3) months (July to September 2019) in three public health facilities in the NR of Ghana. Introductory letters for this research from the Northern regional health administration of the Ghana Health Service were sent to medical superintendents of the selected hospitals offering both TB and DM services in the same facility. This was done after the study had obtained ethical clearance from the University of KwaZulu-Natal (UKZN) Biomedical Research Ethics Committee (BE262/19) and Ghana Health Service Ethics Review Committee (GHS_ERC 012/04/19). Superintendents of the selected health facilities, in turn, gave permission letters and introduced the researcher to the staff of the TB and DM units. The researcher introduced the study to the potential participants and sought their informed consent, prior to discussing and agreeing on the interview dates and time. The written information and informed consent form contained the aim of the study, voluntariness and confidential nature of participation, special permission for audio-recording and the fact that additional hand-written notes were going to be taken during the interviews. Furthermore, potential participants were assured that they could opt out of the study at any point and that would not have any consequences. The whole consent process was explained by the researcher who also invited questions for further clarification. The informed consent form was signed by all potential participants prior to participating in the study. The participants’ names were kept anonymous by referring to them using codes, which further ensured that the confidentiality was maintained.

A semi-structured interview guide developed by the researcher was used to conduct face-to-face in-depth interviews with twenty-three (23) healthcare workers. The interview guide contained open ended questions which allowed the researcher to probe further. Topics covered in the interview guide included: the roles of healthcare workers, knowledge/awareness of TB/DM comorbidity and collaborative framework, treatment guidelines for TB and DM, and the process followed in implementing the bidirectional screening. The healthcare workers interviewed included 3 doctors, 11 nurses, 1 prescriber(A nurse qualified to run consultations and clinics in place of a doctor for some conditions.), 2 health managers, 3 TB institutional coordinators and 3 TB task-shifting officers. Interviews were conducted in English and lasted for 45 minutes to an hour. A suitable location for in-depth interviews was selected by each potential respondent, where they were comfortable and could speak freely.

One hour-long non-participant observations of the screening process were conducted over a two-day period by the researcher in each facility during DM and TB clinic days at the three participating facilities. The researcher also requested the documents on guidelines used in the course of their work. Documents from four categories, namely: screening tools, treatment guidelines, recording forms, and reporting templates (monthly, quarterly, annual reporting templates), were retrieved and reviewed.

### Data analysis

Interviews were audio-recorded, and complemented with hand-written field notes, especially to capture non-verbal data, but also served as a back-up in the event of audio-recording failure. The hand-written notes were taken by the researcher. Twenty-one (21) out of a total of twenty-three (23) participants agreed to the audio recording of interviews. All audio-recorded interviews were later transcribed verbatim by an experienced professional transcriber. Field notes for the two interviews not recorded were written by the researcher. The grounded theory approach was used to guide the data generation and analysis process [20, 21]. Transcripts were systematically reviewed while listening to the audio recordings. Repeated ideas and perspectives were identified and coded. Similar codes were grouped to identify key themes. Themes were triangulated with the document review and observational data and further categorised into facilitators and barriers of the screening process. The observational data allowed the researcher to understand the context of the bidirectional screening phenomenon, which was key for understanding and interpreting data generated. Document review gave valuable information on the guidelines followed by healthcare workers in delivering care.

## Results

A total of twenty-three respondents from three health facilities participated in this study, namely: 9, 6, and 8 from Hospital X, Hospital Y and Hospital Z, respectively (Table 1). Respondents’ ages ranged from 27 to 58 years (mean age = 40.7 years). Study participants comprised of males (n = 14) and females (n = 9). The respondents’ roles varied, including nurse-TB care (n = 6), nurse-DM care (n = 5), task shifting officer-TB care (n = 3), institutional coordinator-TB care (n = 3), hospital manager (n = 2), medical doctor-DM care (n = 2), medical doctor-TB care (n = 1) and nurse prescriber-DM care (n = 1) (Table 1).

**Table 1:** Profiles of the research respondents in the facility, by role, gender, age range and facility.

Hospital X*				Hospital Y*				Hospital Z*			
n	Role	Gender	Age range	n	Role	Gender	Age range	n	Role	Gender	Age range
1	Nurse-TB care	F	35–44	1	Task shifting officer-TB care	M	35–44	1	Hospital manager	M	45–54
2	Nurse-TB care	M	35–44	2	Institutional coordinator-TB care	F	55–64	2	Institutional coordinator-TB care	M	55–64
3	Nurse-TB care	M	35–44 36	3	Nurse-TB care	F	45–54	3	Nurse-TB care	M	25–34
4	Task shifting officer-TB care	F	25–34	4	Medical Doctor-DM care	M	35–44	4	Task shifting officer-TB care	F	25–34
5	Nurse-DM care	F	25–34	5	Nurse-DM care	F	25–34	5	Nurse-TB care	F	25–34
6	Nurse-DM care	F	35–44	6	Hospital manager	M	55–64	6	Nurse-DM care	M	35–44
7	Medical Doctor -DM care	M	55–64					7	Nurse-DM care	M	25–34
8	Medical Doctor -TB care	M	35–44					8	Nurse Prescriber-DM care	M	45–54
9	Institutional coordinator-TB care	M	25–34								

* Names of the hospitals have been withheld to protect their identities.

Six major themes were identified from the data analysis, drawing on all data sources. Two of the themes (increase in staff capacity and institutionalization of bidirectional screening) were facilitators, whereas four (delays in screening, fear and stigmatization of TB, poor collaboration between TB and DM units, and skewed funding for screening) were barriers (Table 2).

**Table 2:** Summary of facilitators and barriers to bidirectional screening.

Facilitators	Barriers
➢ Increase in Staff Capacity	➢ Delays in Screening
➢ Institutionalization of Bidirectional Screening	➢ Fear and Stigmatization of TB
	➢ Poor Collaboration Between TB and DM units
	➢ Skewed Funding for Screening

## Facilitators of bidirectional screening

### Increase in staff capacity

There was a convergence of views from most respondents in asserting that the creation of the “TB task-shifting officer” role promoted the implementation of bidirectional screening. The Ghana National Tuberculosis Control Program (NTP) instituted this new role to boost staff capacity, as shared by one respondent:

“… *they train some people; they call them task-shifting officers*, *and what they do is that they actively look out for the cases*. *When you go to the OPDs(Out-Patient Departments)*, *they are there*. *They administer the screening tool*. *If you go to most of the hospitals*, *they are sitting by the desk where they are checking the vital signs of the patients they administer that questionnaire”*. ***Male- hospital manager***

“*Before we had the TB Task- shifting officers, the numbers were very low. We were missing so many of them but since this active screening, it identified more cases”. **Female institutional coordinator, TB care***

According to the interviews, the TB task-shifting officer is responsible for screening all people visiting the health facility for TB. The screening is conducted at the OPD and specific clinics: Diabetes mellitus Clinic, Antenatal Clinic and the Wards. This TB task-shifting officer is responsible for administering a screening tool and ensuring that samples for suspected cases are taken to the lab for testing. They are also responsible for following up on the patients’ results and to ensure that patients are put on appropriate medication. Weekly and monthly reports of numbers screened and confirmed cases are prepared and submitted by the TB task-shifting officer to the Institutional Coordinator, then to the district health information management system (DHIMS),an electronic national database of the Ghana Health Service as well as the National TB control program database. This was noted through document review by the researcher and interviews.

### Institutionalisation of bidirectional screening

The screening processes, and discrepancies between guidance and practice, became clearer through the course of this research. Healthcare workers in TB and DM exhibited adequate knowledge on the roles and routines for detecting TB among DM. All health facilities in this study had established some referral systems and guidelines between the TB and DM care units. Though these policies exist there was little guidance found within clinic documents for the operationalization of screening TB patients for DM. This was determined through document reviews and observation.

This was substantiated during the interview process, as a task-shifting officer said:

“…*Any suspected case, then they call me. The nurses also refer suspected people to me for screening. After screening, they go to the public health lab with the sample to be tested by the Gene Xpert machine”. **Female TB Task shifting officer***

Another respondent indicated:

“… *We have the forms here, so any patient we suspect, we make a request to the lab when it is confirmed we have a TB unit where they are referred”. **Male nurse prescriber DM care***

However, the process of screening for DM among TB patients was not as well defined as that of screening for TB among DM patients. TB health workers did not actively screen their patients for DM, therefore, any detection of DM was purely accidental. Instead, they focussed efforts on screening for HIV. Further review of documents showed that there was comparatively more documentation and guidance for TB screening among DM patients than DM screening among TB patients, or concurrent screening for TB and DM.

“…*Truth must be told, the only condition we test you for is HIV. Even the NTP who says they are looking for all these things like diabetes haven’t created a column where you test the TB patient for random blood sugar so that if it’s high, you will suspect the person has diabetes or whether the person is a known diabetic, no column to fill that… Sometimes it’s through the history that the patients mention that I have diabetes. Even when they say, we don’t record those as known diabetic, so we don’t have that statistics. For instance, every TB patient is counselled and tested for HIV, and we link them up for care, but we don't test for diabetes”. **Female nurse TB- care***

“*The nurses also screen for other conditions like HIV, which I know they do, but for diabetes, I can’t talk about that”. **Male medical doctor TB care***

## Barriers to bidirectional screening

### Delays in the screening process

The time taken by additional screening was viewed as an obstacle to successful implementation of the bidirectional screening processes, as this negatively affected the time duration a patient would normally spend at the hospital. Furthermore, this screening has not been fully incorporated in the triage routines at the OPD, therefore healthcare workers perceive it as a supplementary service.

“…*At the OPD, we are having challenges. The directive was to screen everyone, it is supposed to be mandatory, but the patients are in a hurry and think it is a waste of time”. **Female TB task-shifting officer***

This situation was also observed by the researcher during clinic days. Patients came as early as 6 am to pick folders, join the queue and began the process to see the doctor. This would ordinarily take an average of 4–5 hours spent at the facility, at a minimum, where an average of 40 patients are seen on clinic days and in some facilities as high as 70 patients. The screening process usually took an average of 10 mins depending on the answers however any delay by the screening sometimes affected patients position in the queue and they expressed frustration at this.

A review of the screening tool by the researcher showed it contained initial questions on symptoms of TB with yes or no answers. Follow up questions were determined by patient’s response, to determine a suspected case or not.

“…*the screening tool is a series of question just simple ones. Whether you are coughing which is the main sign of the pulmonary TB, whether you sweat in the night and other symptoms, so those that they presume they have TB, they immediately request the laboratory test for them”. **Male- hospital manager***

### Fear and stigmatization of TB

Fear and stigmatization of TB, which affected both patients and healthcare workers who work with TB patients, was another very important barrier to achieving bidirectional screening. All respondents in TB care in all the three public health facilities participating in this study agreed that stigma was a serious challenge to the ideals of bidirectional screening.

“… *Even the health staff are scared of TB”. **Male TB task-shifting officer***

“…*You can’t do away with the element of stigmatization among staff. The moment they hear this is a suspected case of TB, some will intentionally not go close”. **Male nurse TB care***

There were reports of patients being treated differently in the event of suspected TB infection. Non-TB health care workers were hesitant to continue care even if the patient had other conditions.

“*The major thing is the other non-TB working staff, their knowledge on TB is an issue because once a person has TB, nobody wants to get closer and the stigma associated with it does not help the work. The patient could have other conditions, severe ones that may kill him/her other than the TB. It could be managed just for an example something medical, maybe the sugar level is so high that medical ward may need to admit, but once the report comes and they have TB, they say come and pick your patient. If you talk with the nurses, they will also tell you the same thing, it’s something that is running that we all know”. **Male medical doctor TB care***

### Poor collaboration between TB and DM units

This study found poor collaboration between the two units at different levels of service delivery, despite the inclusion of screening guidelines and recommendations within formal clinic policies and documents. Administratively, the two units are managed under two different divisions in the health facilities studied. The TB unit is placed under the infectious disease division and the DM under public health division. There is very little linkage or joint activities between these divisions at the hospital levels. There was little, or no joint trainings organised for TB and DM staff to address the TB-DM comorbidity. The poor collaboration between healthcare workers in both the TB and DM units was found to be negatively affecting the identification of TB-DM patients.

“…*No, we do not have any collaboration with the TB unit, we have not had any meetings in that direction”. **Male Nurse DM Care***

“…*We used to have trainings for both our staff and staff of other units including diabetic staff, but for the past two to three years now, there has not been any effective in-service training concerning TB. NTP will tell you they don't have money, but a while back before our time they used to organize routine training, but some of those staff have gone on retirement, some transfer so the knowledge is going down and the willingness too is not there”. **Female nurse TB care***

Some responses pointed to the perception that DM staff did not consider bidirectional screening their responsibility.

“*TB unit working with other units is always a challenge. They see the work like it is different from any other health condition. The same way when we go for a workshop, they say send the TB screening tool to the diabetic unit so that they will screen their patients. They won’t do it”. **Female nurse TB Care***

Another response was:

“*We need every health care worker to take on the role of screening as theirs. If you see someone coughing, it is very simple to pick a form and refer them to the lab. If every healthcare worker would accept these responsibilities, the work will go on well. They should make it like malaria test when someone comes to the consulting room and complains of coughing just get the sputum and refer them to the lab”. **Male TB task-shifting officer***

### Skewed funding for screening

Screening DM patients for TB is supported by the NTP policy’s Intensified Case Finding Initiative (ICF), as shared by participants. Additional staff (TB task-shifting officer) needed to implement this, is funded by the NTP program. The NTP program funds the cost of TB care continuum in its entirety, from laboratory test until patients receive their medication. This study found that general screening for DM in the three public health facilities was limited because hospitals were supposed to provide test strips, but when these were not available, patients had to pay for strips provided by the nurses. Respondents shared what happens as they screen patients:

“*The challenges are so many, because the hospital does not provide strips, we buy from outside, so we have to sell to the patients. The glucometer has been given to us free by a company, but no strips are added. Some of them find it difficult paying, and some of them get annoyed for buying”. **Female nurse DM care***

Another nurse in diabetes care said:

*“Hmmm*, *it is not for free*, *actually*. *The hospital does not provide the strips; patients are supposed to pay for the strips*. *It’s not a force; if you are able to pay for it*, *then they will screen you”*. **Female nurse DM care.**

## Discussion

The TB-DM collaborative framework recommends active screening of TB and DM patients and encourages further research to inform adaptation to local health care systems [3]. The aim of this research was to explore the barriers and facilitators to bidirectional screening in healthcare facilities in the Northern Region of Ghana. This study found that implementing bidirectional screening in public health facilities was achievable, when properly implemented, which is consistent with the findings of similar studies[6, 22, 23]. An increase in staff capacity and institutionalization of bidirectional screening were found to be enablers to the screening process, while delays in screening, fear and stigmatization of TB, poor collaboration between TB and DM units, and skewed funding for screening, were all found to hinder the successful implementation of bidirectional screening in the healthcare facility setting in the Northern Region of Ghana.

The study uncovered several gaps in the implementation process of bidirectional screening in Ghana. In particular, screening to detect TB among DM patients was more organized and focused than screening to detect DM among TB patients. Structures have been put in place, and personnel trained to implement this intervention. However, the reverse of screening TB patients for DM has not been institutionalized. This may be attributed to the little guidance on how to operationalise the policy. Screening TB patients for DM was found to be limited at the health facility level in the Northern Region of Ghana. There seems to be a stronger case made for screening DM patients for TB than TB patients for DM, which is similar to the findings by Yorke et al [24]. A number of reasons could underlie this finding. For example, dedicated funds are not allocated to support the screening of TB patients for DM, and patients end up having to pay for blood sugar strips, unlike in the case of TB screening, which is a free service for all patient. This was found in Ethiopia, where DM patients’ inability to pay for service was a barrier to the co-management of TB-DM [25]. A review on co-management of TB-DM in LMICs also confirmed that funding for DM diagnosis and management is very low, and the cost of this health service to the patient is substantial [26]. The low prioritization of funding support for DM identified in this research is supported by a previous study conducted in Ghana, which revealed that funding is skewed towards infectious diseases including TB and HIV, as compared to non-communicable diseases such as diabetes [27]. With the low priority for the screening of TB patients for DM, Ghana may be having a substantial number of undiagnosed TB-DM cases passing through the public health facilities, undetected. Poor collaboration by healthcare workers of TB and DM units may also weaken bidirectional screening efforts, as was evident in this study and compares well to the findings of other research [28].

This study also found that the introduction of TB task- shifting officers by the NTP, to support existing staff, improved the process of screening DM patients for TB. The global dearth of healthcare workers has been well documented, especially with health systems in LMICs [29]. The introduction of task- shifting is aimed at helping with the shortage of healthcare workers in order to address high workloads [30]. In Ghana, task shifting is not a new phenomenon, and a similar system was seen when the medical assistant's roles were instituted to augment healthcare delivery [31]. Furthermore, the healthcare workers in this study viewed screening as an added responsibility to their already high workload, as was also found in a similar study in Ethiopia[32].

Indications from observations and interviews for the present research findings showed that there were delays in the screening process at the public health facilities. The process of screening patients for TB is an added routine at the designated clinics (OPD, diabetic clinics, antenatal, and wards). Patients were reported to find it time-consuming, and non-TB healthcare workers found it disruptive to their routines. Another study conducted in Ghana attributed low TB case detection to delay in patients’ health-seeking behaviour, but this study found delays in screening process at the health facility level to be another factor for low TB case detection, as a result, patients end up opting-out of screening [25, 33, 34].

Finally, the study highlights that stigma continues to exist and hinder TB screening and care, despite the many years of health advocacy and awareness programme implementation [34]. A paper on TB stigma in Ghana came to the same conclusion as our findings on TB stigma among health care workers [35]. These findings from our study provides useful evidence to support the implementation of bidirectional screening for improved outcomes in TB and DM.

Our study has some limitations. First, the viewpoint of patients was missed, and further studies on patients’ barriers and enablers for bidirectional screening is recommended. Second, our sample includes only three of several facilities in NR. Nonetheless, some important contextual considerations to bidirectional TB and DM screening were identified. Additionally our study design did not allow for estimating prevalence of TB among DM patients and vice versa in the selected health facilities however interviews from healthcare workers gave an indication on the state of TB-DM comorbidly detection.

## Conclusions

This study found that bidirectional screening at the three public health facilities in Northern Ghana is being implemented; operationalising this policy has been enhanced by the improved staff capacity, funding, and institutionalization. However, some barriers to optimal implementation continue to exist. To improve screening outcomes and overcome the barriers in LMICs such as Ghana, special attention will need to be given to screening TB patients for DM, and developing a cost-effective screening approach for DM that is comparable to the current TB screening approach in order to help identify high-risk DM individuals who can then move on to be tested. There still exist gaps in knowledge of the TB-DM comorbidity and management among healthcare workers, thereby supporting the case for initiatives that will strengthen TB-DM collaboration. Additionally, there should be health education programs to create awareness and help patients appreciate the value of having few extra minutes for additional screening.

## Supporting information

S1 Checklist (DOCX)

S1 File (PDF)

S2 File (PDF)

## List of abbreviations

DHIMS: District Health Information Management System
DM: Diabetes Mellitus
GHS: Ghana Health Service
ICF: Intensified Case Finding
IUALD: International Union against Tuberculosis and Lung Disease
LMICs: Low-and Middle-Income Countries
MOH: Ministry of Health
NR: Northern Region
NTP: National Tuberculosis Control Programme
OPD: Out- Patient Department
TB: Tuberculosis
WCA: Western and Central Africa
WHO: World Health Organisation
