# Supplementary material for: Barriers and facilitators to bidirectional screening of TB-DM in Ghana: Healthcare workers’ perspectives
Source: PLoS One. 2020 Jul 14;15(7):e0235914. doi: 10.1371/journal.pone.0235914 (PMC7360027; doi:10.1371/journal.pone.0235914)
Supplement: S1 Checklist — (DOCX) [file pone.0235914.s001.docx]

COREQ checklist Consolidated criteria for reporting qualitative studies (COREQ): 32-item checklist

| **No. Item** | **Guide questions/description** | **Reported on Page #** |
| --- | --- | --- |
| **Domain 1: Research team and reﬂexivity** | | |
| *Personal Characteristics* | | |
| 1.Interviewer/facilitator | Which author/s conducted the interview or focus group? | **Rita Suhuyini Salifu** |
| 2. Credentials | What were the researcher’s credentials? | **Rita Suhuyini Salifu**  BSc. Comm. Nutrition.  MA. International Health and currently a Ph.D. Candidate.  **Dr. Khumbulani Welcome Hlongwana: PhD, MPH, MA, BAHons.**  Senior Lecturer. |
| 3. Occupation | What was their occupation at the time of the study? | Student  Senior lecturer |
| 4. Gender | Was the researcher male or female? | Female |
| 5. Experience and training | What experience or training did the researcher have? | The researcher participated in qualitative research methods training at the University of Copenhagen in 2013. The researcher conducted a qualitative study for her master’s dissertation in 2014. She also participated in research workshop incorporating qualitative research at the University of KwaZulu-Natal in 2019. |
| 6. Relationship established | Was a relationship established prior to study commencement? | Except for introductory meetings, no solid relationship was established prior to study commencement. |
| 7. Participant knowledge of the interviewer | What did the participants know about the researcher? e.g. personal goals, reasons for doing the research. | Information about who the researcher was and the purpose of the research were contained in the Participant information sheet and Consent form. |
| 8. Interviewer characteristics | What characteristics were reported about the interviewer/facilitator? e.g. Bias, assumptions, reasons and interests in the research topic. | Apart from her student status, not much was reported. |
| **Domain 2: study design** |  |  |
| *Theoretical framework* |  |  |
| 9. Methodological orientation and Theory | What methodological orientation was stated to underpin the study? e.g. grounded theory, discourse analysis, ethnography, phenomenology, content analysis. | The study was an exploratory qualitative research and the grounded theory approach was used to guide data generation and analysis process (Pg 4) |
| *Participant selection* |  |  |
| 10. Sampling How were participants selected? | e.g. purposive, convenience, consecutive, snowball | This study used a heterogenous purposive sampling technique to select participants ( Pg. 4) |
| 11. Method of approach | How were participants approached? e.g. face-to-face, telephone, mail, email | The researcher was introduced to the staff of the TB and DM units face to face. (Pg. 4) |
| 12. Sample size | How many participants were in the study? | Twenty-three (23) healthcare workers involved in TB and DM care were recruited (Pg. 4) |
| Methods 13. Non-participation | How many people refused to participate or dropped out? Reasons? | None |
| *Setting* |  |  |
| 14. Setting of data collection | Where was the data collected? e.g. home, clinic, workplace. | Health Facilities (Pg. 4) |
| 15. Presence of nonparticipants | Was anyone else present besides the participants and researchers? | Non-participants were not allowed into the interview venue. |
| 16. Description of sample | What are the important characteristics of the sample? e.g. demographic data, date | Participants were health facility managers and front-line healthcare worker in TB and DM departments. Ages ranged from 27 to 58 years comprising of 14 males and 9 females .Specific roles were nurses , task shifting officers , institutional coordinators, hospital managers , medical doctors and nurse prescriber. (Pg 6) |
| 17. Interview guide | Were questions, prompts, guides provided by the authors? Was it pilot tested? | Yes these were provided in the interview guides. The interview guide was not piloted. (Pg. 5) |
| 18. Repeat interviews | Were repeat inter views carried out? If yes, how many? | No repeat interviews were carried out |
| No 19. Audio/visual recording | Did the research use audio or visual recording to collect the data? | Yes, on participant’s permission (Pg. 4). |
| 20. Field notes | Were ﬁeld notes made during and/or after the inter view or focus group? | Yes (Pg. 5) |
| 21. Duration | What was the duration of the interviews or focus group? | 45 minutes to an hour(Pg. 5) |
| 22. Data saturation | Was data saturation discussed? | No |
| 23. Transcripts returned | Were transcripts returned to participants for comment and/or correction | No |
| **Domain 3: analysis and ﬁndings** |  |  |
| *Data analysis* |  |  |
| 24. Number of data coders | How many data coders coded the data? | One (Pg. 5) |
| 25. Description of the coding tree | Did authors provide a description of the coding tree? | No |
| 26. Derivation of themes | Were themes identiﬁed in advance or derived from the data? | Themes were derived from data(Pg. 5 & 7) |
| 27. Software | What software, if applicable, was used to manage the data? | N/A |
| 28. Participant checking | Did participants provide feedback on the ﬁndings? | No |
| *Reporting* |  |  |
| 29. Quotations presented | Were participant quotations presented to illustrate the themes/ﬁndings? Was each quotation identiﬁed? e.g. participant number | Yes- Results(Pg.7-12) |
| 30. Data and ﬁndings consistent | Was there consistency between the data presented and the ﬁndings? | Yes -Discussion. Pg.12-14 |
| 31. Clarity of major themes | Were major themes clearly presented in the ﬁndings? | Yes- Results. Pg 7-12. |
| 32. Clarity of minor themes | Is there a description of diverse cases or discussion of minor themes? | Yes -Results.Pg7--12 |
