# Supplementary material for: Barriers and facilitators to bidirectional screening of TB-DM in Ghana: Healthcare workers’ perspectives
Source: PLoS One. 2020 Jul 14;15(7):e0235914. doi: 10.1371/journal.pone.0235914 (PMC7360027; doi:10.1371/journal.pone.0235914)
Supplement: S2 File — (PDF) [file pone.0235914.s003.pdf]

D. INTERVIEW GUIDE -HEALTH WORKER

Good ……………………. My name is Rita Quist-Therson, a student from School of Nursing and Public Health, University of KwaZulu-Natal. I am currently carrying out a research on the *Barriers and Facilitators to the Implementation of the Collaborative Framework for Care and Control of Tuberculosis (TB) and Diabetes (DM) in Ghana*. You have been invited as a participant in this research and I will like to have a discussion with you concerning this topic. I will need between 45-60minutes of your time. You are free to opt of the interview now or at any point in time you don't feel comfortable or skip questions you don't feel comfortable answering. You can also be rest assured that your identity or name as a respondent will not appear anywhere in the study, it will be kept confidential. I will however take down notes and a digital voice recorder to be able to cross check later with the notes to be sure we captured the right information. However, you are free to reject to any of them or both. Thank you.

Introduction

- Kindly share with me your current role in this health facility? For example, describe your typical day at work, what do you do?
- How are the TB/DM clinic sessions organized in this health facility? Please elaborate

Health Care Delivery to TB or DM Patients

- What are the key challenges entailed in your work/ you face during the course of doing your work? Please elaborate
  Probe: *How do you deal with this*?

Knowledge/ Awareness of TB/DM Comorbidity

- How would you describe the condition of DM among TB patients/ TB among DM patients? Please elaborate.
- Please describe the process you follow in this clinic for detecting TB in DM patients? /DM in TB patients?
- Can you share your experiences in managing TB/DM comorbidity in patients?

  Probe: *Please elaborate on some of the issues you face?*
- From your experience, please share how has TB/DM comorbidity impacted on the care and control of TB and DM?

  Probe: *In the area of care pathways at the TB and DM clinics*

  Probe: *Staff capacity/training,*

  Probe: *Work load*
- Kindly elaborate on any recommendations you have that can help improve TB/DM co-management?

Bidirectional Screening

- ❖ Kindly list guidelines /policies guiding detection of DM among TB patients/ TB among DM patients and elaborate on what each entails? (Request for the copies of these)

- ❖ Kindly share about how TB screening among DM patients impacts TB case detection and vice versa? Please elaborate

- ❖ How has the framework impacted your role? Please elaborate

  ***Probe**: Please describe the issues you face in screening?*

- ❖ Kindly share about any collaborative activities between staff in TB and DM clinics?

- ❖ Please describe the process followed in this facility in managing a TB patient if DM is detected and vice versa?

- ❖ Can you share some practices that demonstrate acceptance or rejection of the collaborative framework? Please elaborate

  ***Probe**: In your opinion, are the practices listed above peculiar to only the Northern region? Please elaborate*

- ❖ Please share any recommendations that you think will help improve the TB/DM care and control. Kindly elaborate

- ❖ Please share your advice on what you think will hinder the implementation of the Collaborative Framework? Kindly elaborate

❖ Kindly tell me briefly about yourself

- *Participant code*
- *Age*
- *Educational background/training*

GENERAL IMPRESSIONS

I have attempted to pose some questions in relation to your experience in the implementation of the collaborative framework. I may have not been able to capture all the issues in relation to the subject matter. Is there anything you consider important in this respect but which I did not touch on? Can you shed some light on this?

Thank you for your time. Do you have any questions for me?
